# Machine learning models for mortality prediction in patients with spontaneous subarachnoid hemorrhage following ICU treatment

**DOI:** 10.3389/fneur.2025.1648353

**Published:** 2025-09-17

**Authors:** Wenwen Hu, Danfeng Yu, Liwen Zhang, Jing Zhang

**Affiliations:** ^1^Department of Neurological Intensive Care Unit, Taihe Hospital, Hubei University of Medicine, Shiyan, China; ^2^Graduate School, Hubei University of Medicine, Shiyan, China

**Keywords:** subarachnoid hemorrhage, intensive care unit, machine learning, predictive model, MIMIC-IV database

## Abstract

**Background:**

Spontaneous subarachnoid hemorrhage (SAH) is a severe and potentially life-threatening acute cerebrovascular disease. Early identification of the risk of death in patients with spontaneous SAH is of vital importance for improving prognosis, reducing mortality, and guiding clinical treatment.

**Methods:**

A retrospective cohort study was conducted using the public database, Medical Information Mart for Intensive Care IV (MIMIC)-IV. The primary outcome was in-hospital mortality following intensive care unit (ICU) treatment. All features were extracted from first-day ICU admission data. Data analysis was performed by using R and Python, with feature selection conducted via least absolute shrinkage and selection operator (LASSO) regression. We constructed 8 models based on the 12 selected features in the training set and evaluated them in the test set by various metrics, including area under the curve (AUC), accuracy, precision (positive prediction value), recall (sensitivity), Brier score, Jordan index, and calibration slope. The most effective model was rendered explainable through the SHapley Additive exPlanations (SHAP) approach.

**Results:**

The study included 1,121 records, with 870 surviving and 251 deceased patients. We selected 43 features for the preliminary baseline analysis. Based on LASSO regression analysis and clinical practical significance, 12 features were finally included in the construction of the machine learning models. We constructed eight machine learning models, among which the logistic regression (LR) model performed the best.

**Conclusions:**

In our study, the LR model exhibited superior discrimination in predicting risk of mortality among patients with spontaneous SAH compared to other models. This research contributes to facilitating the early identification of mortality risk in patients with spontaneous SAH. External validation and further prospective studies are warranted to confirm and refine these predictive insights for clinical utilization.

## 1 Introduction

Subarachnoid Hemorrhage (SAH) is a critical public health concern, which remains a serious disease associated with considerable disability and mortality (1). The incidence of SAH is approximately 9 cases per 100,000 individuals, and it is the third most prevalent subtype of stroke (2). This disorder is primarily categorized into two types: traumatic and spontaneous (non-traumatic), with the spontaneous type accounting for approximately 85%−95% of cases, thus constituting the majority (3). Spontaneous SAH is relatively common, and its causes are diverse. The rupture of intracranial aneurysms is one of the main causes, accounting for approximately 85% (4). Cerebral vascular malformations, such as arteriovenous malformations, are also important contributing factors, occurring more frequently in adolescents (5). In addition, vascular inflammation, abnormal vascular networks at the base of the brain, brain tumors, and moyamoya disease may also lead to spontaneous SAH (5).

One-third of spontaneous SAH patients die within the initial days to weeks after the hemorrhage, and most survivors have long-term disability or cognitive impairment (6). Spontaneous SAH carries an exceptionally high disease-specific burden. Since traditional risk prediction is limited to a single feature selection method or a single algorithm, it has relative lag and limitations (7). There is an urgent need for a reliable method to predict the risk of death in spontaneous SAH patients in the ICU at an early stage. Clinical prediction models that utilize electronic health record data through advanced data mining techniques have emerged as a promising approach to addressing these challenges. Machine learning, with its high efficiency and accuracy in data processing, has become increasingly prevalent in various disease predictions. In our study, we aimed to integrate machine learning algorithms with traditional statistical analysis to comprehensively evaluate the risk factors for death in patients with spontaneous SAH following intensive care unit (ICU) treatment. These new analytic approaches may enhance risk prediction beyond only traditional statistical approaches used in the past (8). An assessment of the risk of death after spontaneous SAH is valuable for guiding early clinical management of patients and evaluating clinical efficacy.

## 2 Methods

### 2.1 Data source and study population

This study is a retrospective cohort study based on the Medical Information Mart for Intensive Care IV (MIMIC-IV, Version 3.1, released on 11 October 2024) database (9). In order to enhance usability of medical data and to improve patient care through knowledge discovery and algorithm development, a large deidentified dataset - MIMIC-IV database, developed and maintained by the Computational Physiology Laboratory at the Massachusetts Institute of Technology (MIT). MIMIC-IV contains data for over 65,000 patients admitted to an ICU and over 200,000 patients admitted to the emergency department at the Beth Israel Deaconess Medical Center in Boston, MA. All data was captured automatically through the three systems during clinical care: Hospital-wide Electronic Health Record (EHR) System, ICU Clinical Information (MetaVision) System, and Emergency Department (ED) System.

For data retrieval from the MIMIC-IV database, Structured Query Language (SQL) was applied. In order to comply with the regulations, the author, Wenwen Hu, obtained both a Cooperative Institutional Training Initiative (CITI) license and the necessary permissions to use the MIMIC-IV database (ID: 67812003). We developed detailed data extraction steps and conducted trial extractions before the official data extraction phase to test and refine the clarity and operability of these steps.

(1) Inclusion criteria
  a. Patients who were diagnosed with spontaneous SAH confirmed by both the International Classification of Diseases (ICD)-9 or ICD-10.
  b. For patients with ICU admissions more than once, only data of the first ICU admission of the first hospitalization was collected for the study.
(2) Exclusion criteria
  a. Patients under the age of 18 were excluded from the study.
  b. Patients with concurrent malignant tumors were excluded from the study.
  c. Patients with over 20% missing features (after feature extraction) were also excluded from the study.

### 2.2 Feature selection and outcome

In this study, 43 features referring to published articles (10–13) and clinical experience were extracted from the MIMIC-IV database, including age, gender, basic vital signs, coexisting disorders, blood cell analysis, coagulation function, serum ions, biochemical parameters, ventilation status, Glasgow Coma Scale (GCS) score, sepsis-related organ failure assessment (SOFA) score, acute physiology score iii (APS III), simplified acute physiology score ii (SAPS II) and the primary outcome.

The vital signs and serum ions were selected based on the maximum and minimum values recorded on the first day of admission to the ICU, while blood cell analysis, coagulation function, liver and kidney function, and serum ions were selected based on the first test values recorded on the first day of admission to the ICU. In cases where multiple test results were available for a specific feature, the first measurement was used in the analysis.

The variance inflation factor (VIF) is an effective tool to detect multicollinearity (14). A VIF = 1 indicates no multicollinearity; a VIF between 1 and 5 indicates moderate collinearity; a VIF > 5 indicates high collinearity; a VIF > 10 indicates severe multicollinearity (15). To mitigate the interference caused by strong multicollinearity, we removed 5 features with severe multicollinearity. Re-calculated the VIF of the retained features, all of them were < 5.

We applied the least absolute shrinkage and selection operator (LASSO) regression in feature selection. LASSO achieves variable selection through L1 regularization. As the value of λ increases, more and more coefficients are shrunk to zero, resulting in twelve features with nonzero coefficients (16). The primary outcome was in-hospital mortality of spontaneous SAH patients following their treatment in the ICU.

### 2.3 Missing data processing

Missing data is inevitable because clinical needs and resources limit what data is collected, patient differences lead to inconsistent or variable measurements, and merging data from various sources may introduces omissions and discrepancies. Most features had missing rates < 10%, with the exception of PT (12.2%) and APTT (13.7%) (Supplementary Table S1).

For data with a missing rate of less than 10%, a filling method (median, mean, or mode) that represents the central tendency of the variables was selected based on the characteristics of the data distribution (17). For data with a missing rate ranging from 10% to 20%, the multiple imputation method was employed to replace missing values, thereby minimizing their impact on classification performance (18).

When the missing rate is low (< 10%), the bias introduced by simple imputation is typically negligible compared to the additional complexity associated with multiple imputation. Multiple imputation is designed for higher missing rates. In our study, simple imputation and multiple imputation didn't make significantly different results. Variability and the relationships between variables were preserved as they would be when using multiple imputation.

### 2.4 Statistical analysis

Categorical variables were presented as numbers and percentages (%). We compared proportions for unordered categorical variables using the χ^2^-test or Fisher's exact test and compared proportions for ordered categorical variables using the Wilcoxon rank-sum test. Normally and non-normally distributed continuous variables were expressed as mean ± SD and median (interquartile range, IQR), respectively. Normally distributed continuous variables were analyzed by an independent *t*-test, while non-normally distributed continuous variables were analyzed by the Mann–Whitney U test. *P* values less than 0.05 (two-sided test) were considered statistically significant.

### 2.5 Model construction and evaluation

Model construction was performed using 8 machine learning algorithms, including Random Forest (RF), Logistic Regression (LR), Light Gradient Boosting Machine (LGBM), Naive Bayes (NB), Decision Tree (DT), Extreme Gradient Boosting (XGBoost), Support Vector Machine (SVM), and Artificial Neural Network (ANN). The performance of each model was evaluated depending on area under the curve (AUC), accuracy, precision, recall, Brier score, Jordan index, and calibration slope. Receiver operating characteristic (ROC) curves and precision–recall (P-R) curves for the eight models were depicted in one plot, respectively, for comparison. The metrics and plots were used to determine the optimal model. Additionally, the SHapley Additive exPlanations (SHAP) approach was adopted to make the final optimized model more interpretable. The SHAP values indicate the contribution of each feature to the final classification, enabling us to interpret the model from a clinical perspective.

### 2.6 Software

We used Navicat (version 17.0.8) to access the MIMIC-IV database. Data preprocessing, feature selection, and statistical analysis were performed using R (version 4.4.3). Python (version 3.13.1) was employed for the construction and evaluation of machine learning models.

## 3 Results

### 3.1 Baseline characteristics of ICU patients with SAH

**Figure 1:**
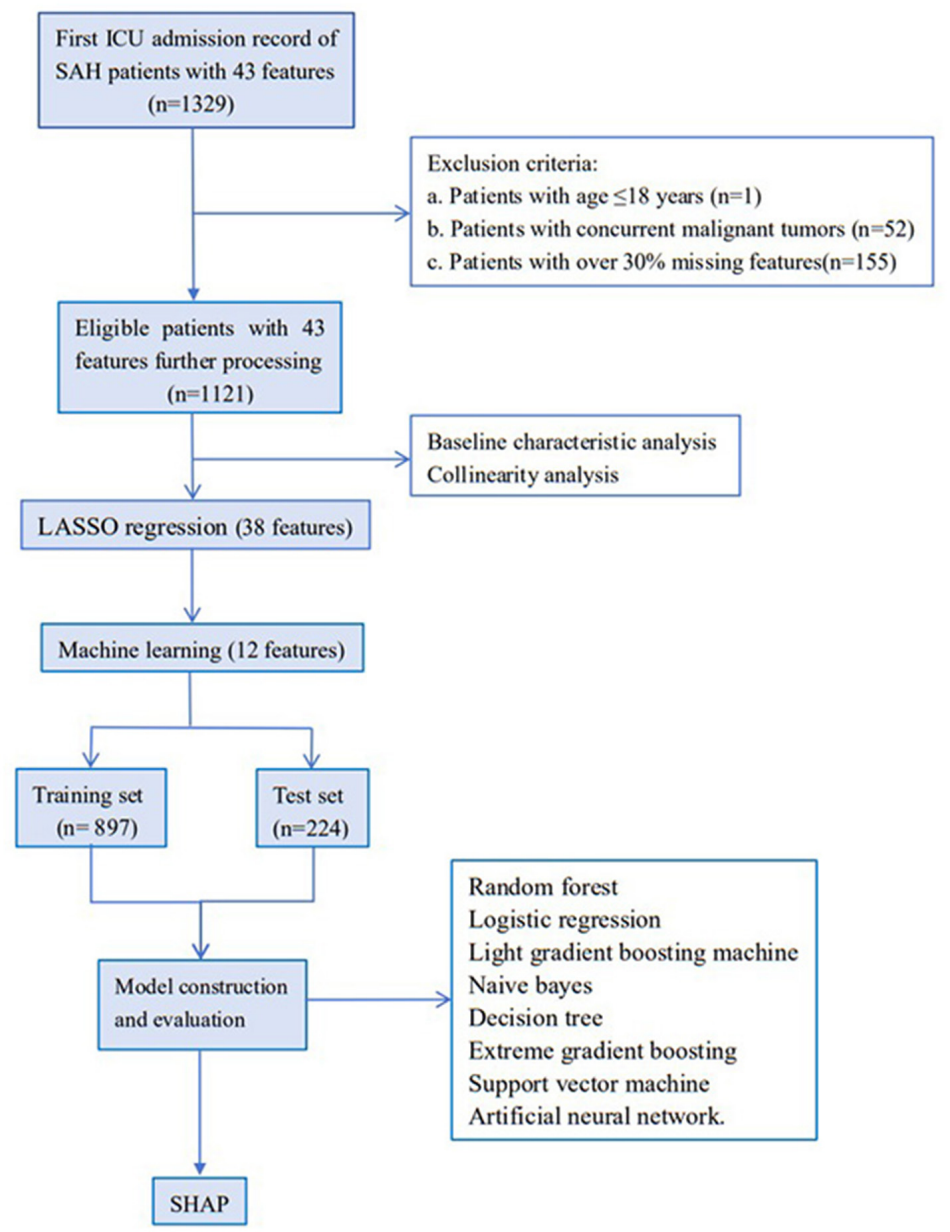
Patients and features selection flowchart of the MIMIC-IV database.

Initially, 1,329 ICU admission records diagnosed with spontaneous SAH and 43 features were extracted from the MIMIC-IV database. By combining exclusion criteria and discarding features with excessive missing values, 1,121 records were ultimately retained for subsequent analysis. The flowchart of the study is shown in Figure 1. Based on ICU outcomes, the entire study population was divided into two groups: a survival group (*n* = 870) and a non-survival group (*n* = 251). Baseline characteristics of the included patients are presented in Table 1.

**Table 1:** Characteristics of spontaneous SAH from the MIMIC-IV database.

**Variables**	**Survival (*n* = 870)**	**Non-survival (*n* = 251)**	***P* Value**
**Baseline characteristics**			
Admission age (years)	59.73 (50.21, 70.64)	69.49 (57.83, 80.94)	< 0.001^*^
**Gender**			0.271
Male	359 (41.26 %)	114 (45.42 %)	
Female	511 (58.74 %)	137 (54.58 %)	
**Vital signs (1st 24 h)**			
Heart rate-max (b.p.m)	96 (85, 106)	103 (92, 118)	< 0.001^*^
NBPS-max (mmHg)	155 (143, 167)	157 (145, 175)	0.004^*^
NMAP-max (mmHg)	82 (75, 88)	83 (76, 91)	0.026^*^
SpO_2_-min (%)	93 (91, 95)	94 (90, 97)	0.877
Glucose-max (mg/dL)	143.21 (122.35, 171.38)	179.03 (145.31, 246.5)	< 0.001^*^
Temperature-max (°C)	37.39 (37.11, 37.78)	37.56 (36.94, 38.30)	0.049^*^
**Coexisting disorders**			
Chronic pulmonary disease	110 (12.64 %)	38 (15.14 %)	0.356
Congestive heart failure	50 (5.75 %)	29 (11.55 %)	0.002^*^
Myocardial infarct	822 (94.48 %)	30 (11.95 %)	< 0.001^*^
Diabetes	115 (13.22 %)	45 (17.93 %)	0.076
Renal disease	40 (4.60 %)	31 (12.35 %)	< 0.001^*^
Liver disease	22 (2.53 %)	25 (9.96 %)	< 0.001^*^
Rheumatic disease	14 (1.61 %)	3 (1.20 %)	0.857
Hypertension	514 (59.08 %)	164 (65.34 %)	0.087
Peptic ulcer disease	6 (0.69 %)	3 (1.20 %)	0.573
Aids	3 (0.34 %)	2 (0.80 %)	0.682
**Smoking and drinking history**			
Alcohol	31 (3.56 %)	10 (3.98 %)	0.903
Tobacco	104 (11.95 %)	29 (11.55 %)	0.951
**Serum ions (1st 24 h)**			
Sodium-max (mEq/L)	140.37 (139.09, 143.25)	144.21 (140,06, 148.50)	< 0.001^*^
Sodium-min (mEq/L)	138.13 (136.09, 140.36)	139.07 (135.21, 141.70)	0.200
Potassium-max (mEq/L)	4.11 (3.81, 4.52)	4.34 (4.02,4.91)	< 0.001^*^
Potassium-min (mEq/L)	3.73 (3.42, 4.28)	3.76 (3.31,4.14)	0.522
Calcium-min (mg/dL)	8.43 (8.08, 8.91)	8.23 (7.75,8.80)	< 0.001^*^
Calcium-max (mg/dL)	8.82 (8.41, 9.23)	8.86 (8.25,9.33)	0.897
**Blood cell analysis (1st 24 h)**			
RBC (K/μL)	4.12 (3.74, 4.51)	4.02 (3.54,4.54)	0.058
Platelet (K/μL)	219.58 (182.25, 267.75)	203.02 (152.50, 262.51)	< 0.001^*^
WBC(K/μL)	11.06 (8.51, 13.93)	13.15 (10.23, 16.251)	< 0.001^*^
Lymphocyte (K/μL)	12.98 (9.02, 16.96)	11.39 (6.28, 14.47)	< 0.001^*^
Hematocrit (%)	37.26 (33.91, 40.34)	36.97 (32.75, 40.45)	0.272
Hemoglobin (g/dL)	12.56 (11.31, 13.64)	12.42 (10.85, 13.50)	0.163
**Coagulation function (1st 24 h)**			
PT(s)	12.33 (11.52,13.24)	12.70 (11.62,13.91)	< 0.001^*^
APTT (s)	27.91 (25.41,30.82)	27.42 (24.85,31.53)	0.539
INR	1.13 (1.04,1.25)	1.21 (1.10,1.38)	< 0.001^*^
**Renal function (1st 24 h)**			
Creatinine-max (mg/d)	0.84 (0.71,1.03)	1.07 (0.81, 1.42)	< 0.001^*^
BUN-max (mg/dL)	15.06 (12.15, 19.23)	20.27 (15.18, 28.04)	< 0.001^*^
**Scoring system (1st 24 h)**			
GCS-min	13 (10, 15)	8 (3, 15)	0.009^*^
SOFA	2 (1, 4)	4 (2, 7)	< 0.001^*^
APS III	30 (23, 40)	47 (32, 67)	< 0.001^*^
SAPS II	27 (22, 36)	39 (34, 52)	< 0.001^*^
Ventilation	644 (74.02%)	221 (88.05%)	0.254
Return ICU	92 (10.57 %)	24 (9.56 %)	0.729

The unit of measurement is extracted from MIMIC-IV database without any changes.

b.p.m, beats per minute; NBPS, non-invasive blood pressure systolic; NMAP, non-invasive mean arterial pressure; SpO_2_, saturation of pulse oxygen; RBC, red blood cell count; WBC, white blood count; PT, prothrombin time; APTT, activated partial thromboplastin time; INR, international normalized ratio; BUN, blood urea nitrogen; GCS, glasgow coma scale; SOFA, sequential organ failure assessment; APS III, Acute Physiology Score III; SAPS II, Simplified Acute Physiology Score II; ICU, intensive care unit.

^*^P values less than 0.05.

Forty-three characteristics were compared between the two groups, among which 24 characteristics showed significant statistical difference. According to the baseline data presented in the Table 1, patients in the non-survival group were generally older than those in the survival group. No significant difference was observed in gender composition between the two groups, with females slightly outnumbering males in both the survival and non-survival groups. The data revealed that the non-survival group was more likely to have electrolyte disorders, coagulation abnormalities, hyperglycemia, and thrombocytopenia. Additionally, vital signs such as heart rate, body temperature, and oxygen saturation in these patients fluctuated more widely and were more likely to be accompanied by comorbidities.

### 3.2 Features selection

Given that the red blood cell (RBC) count influences hemoglobin and hematocrit levels, RBC was retained. The calculation of non-invasive mean arterial pressure (NMAP) is dependent on non-invasive systolic blood pressure (NBPS); hence, NBPS was retained. Given the close interrelationship among INR, PT, and APTT, APTT was retained. Consequently, five features - hemoglobin, hematocrit, INR, PT, and NMAP - with excessive multicollinearity were removed, and 38 features were retained for further analysis (Supplementary Figure S1).

**Figure 2:**
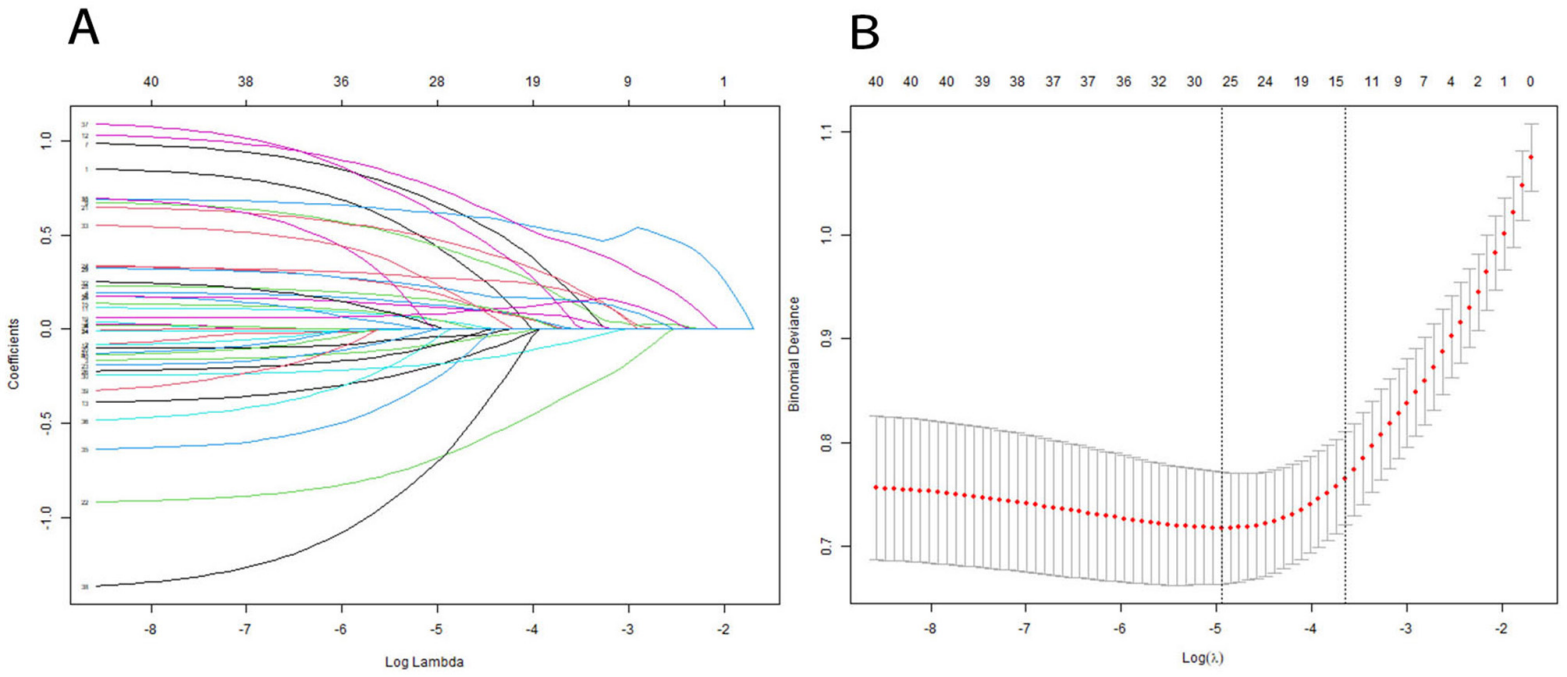
Features selection using a LASSO regression model. **(A)** LASSO coefficient path graph: Lasso achieves variable selection through L1 regularization. As the value of λ increases, more and more coefficients are shrunk to zero. To determine the optimal predictors of the model, ten-fold cross-validation with minimum criteria was used, resulting in twelve features with nonzero coefficients. **(B)** The minimum criteria (lambda.min) and 1 SE of the minimum criteria (lambda. 1se) were used to depict the optimal values with dotted vertical lines. We chosed lambda.1se instead of lambda.min because lambda.1se (the maximum λ value within the minimum error range of one standard error) usually provides a more robust and concise model, which helps to avoid overfitting.

**Figure 3:**
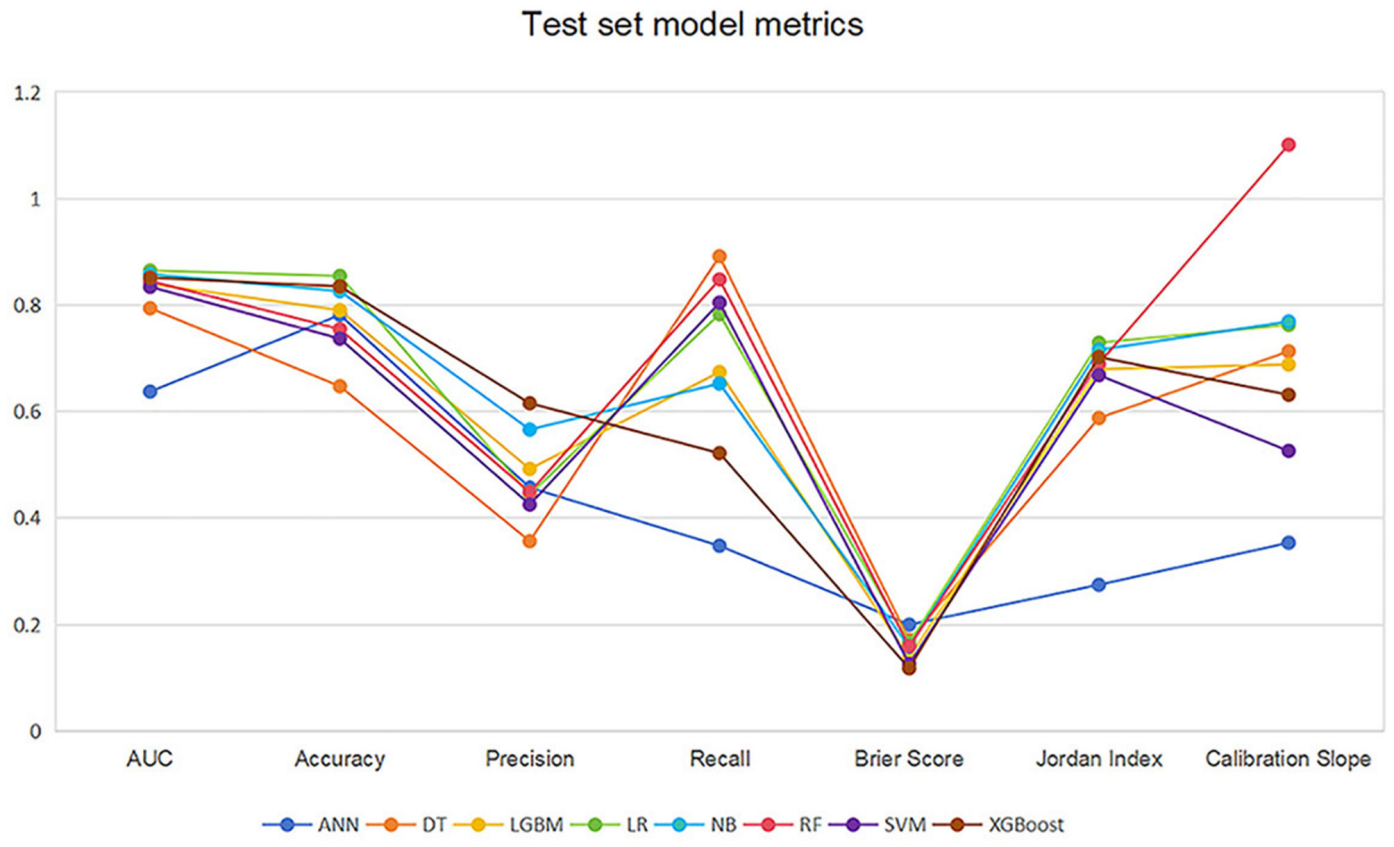
LASSO coefficient profile of 12 features. Features after selection. There are 38 features in total before the selection process, and 12 features remain after using LASSO regression. The plot presents the top 12 features that had the greatest impact on survival or death in SAH patients after receiving treatment in the ICU. Green bars indicate protective factors and red bars indicate risk factors. The length of the bar for each feature indicates the importance (weight) of that feature in making the prediction. A longer bar indicates a feature that contributes more to survival or death.

Subsequently, the retained features were selected by the LASSO regression algorithm. Twelve of 38 features were selected as the best predictive to construct the machine learning models. These were identified at a shrinkage parameter (lambda.1se) of 0.02618559 (Figure 2). The following features raise the risk of mortality in our study: serum sodium, SAPSII score, admission age, BUN, glucose, SOFA score, heart rate, APSIII score, liver disease, and creatinine. Conversely, when SpO2 and platelet count rise, the risk of death falls. The importance ranking of the 12 features is shown in Figure 3. Then, these features were used in the subsequent analyses for all models in both training and test sets.

### 3.3 Model performance and explanation

1,121 records were randomly divided into a training set (*n* = 897) and a test set (*n* = 224) at a ratio of 8:2. The number of non-survival patients was 205 (22.9%) and 46 (20.5%) in the training and test sets respectively. We developed 8 machine learning models to predict the risk factors for death after receiving treatment in ICU. The 8 models were trained employing the training set. Their performance was subsequently evaluated employing the test set.

**Figure 4:**
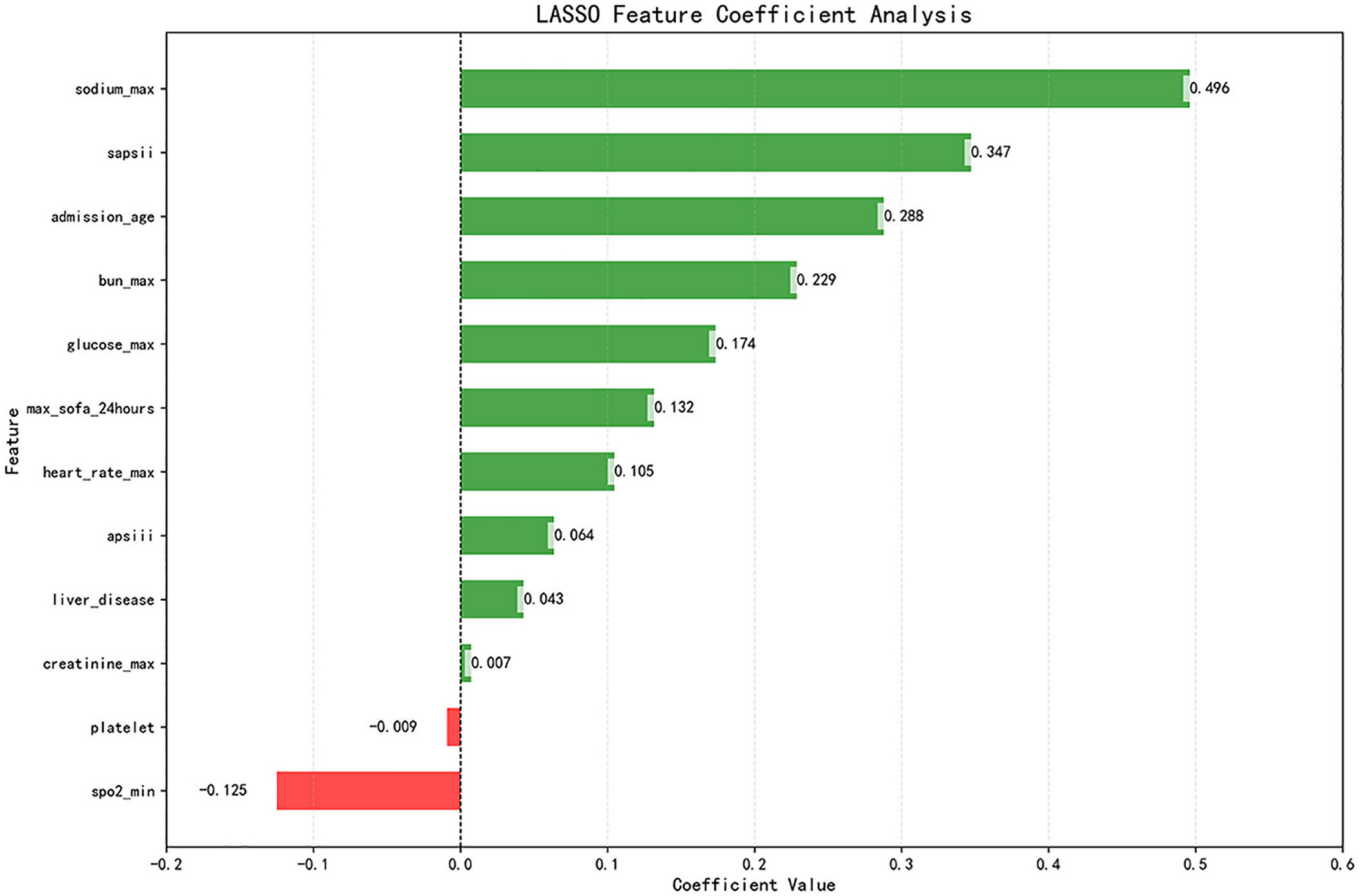
Line graph of the test set model metrics. LR, logistic regression; RF, random forest; LGBM, light gradient boosting machine; NB, naive bayes; DT, decision tree; XGBoost, extreme gradient boosting; SVM, support vector Machine; ANN, artificial neural network.

**Figure 5:**
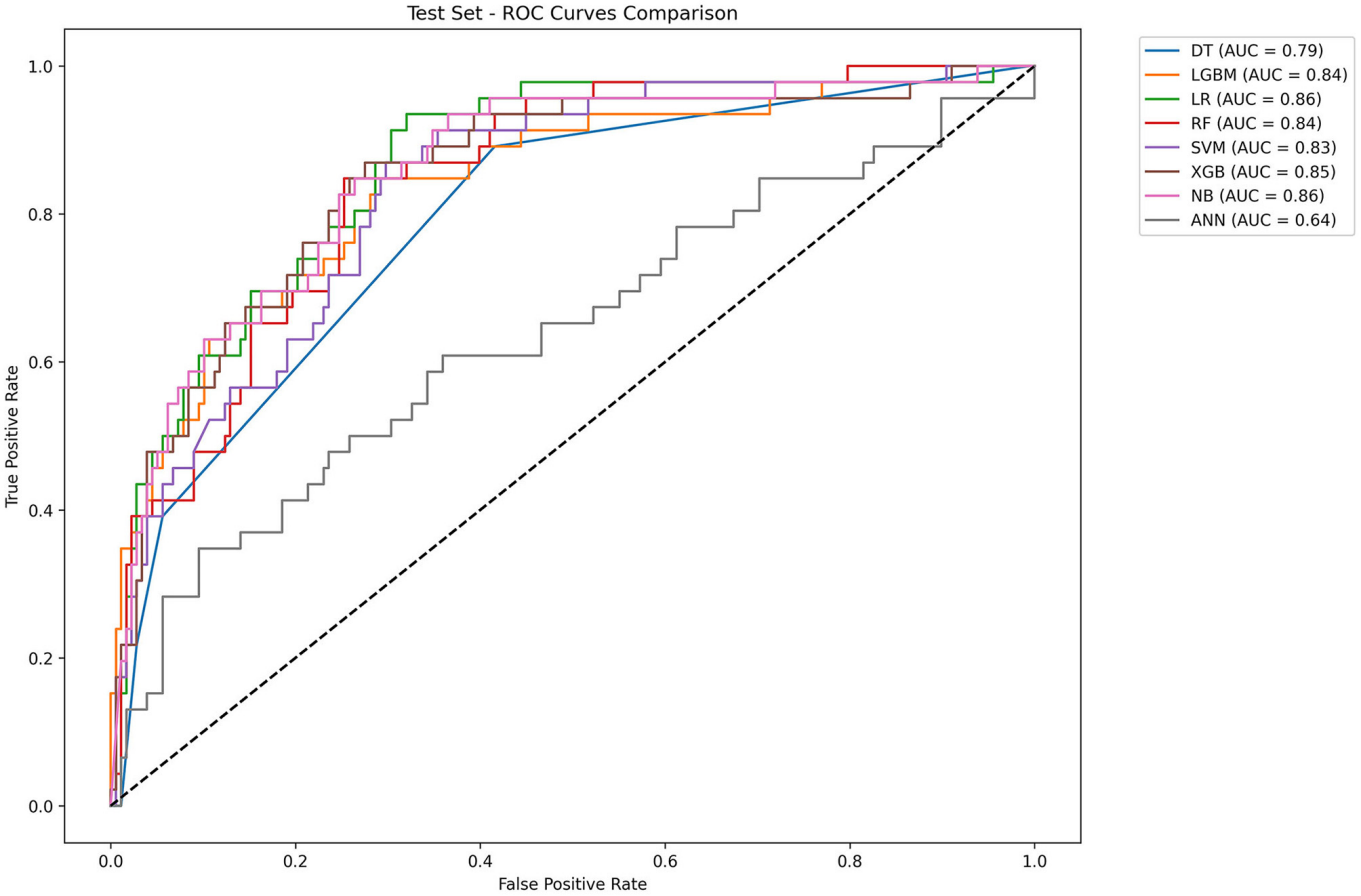
ROC curves of eight machine learning models. LR, logistic regression; RF: random forest; LGBM, light gradient boosting machine; NB, naive bayes; DT, decision tree; XGBoost, extreme gradient boosting; SVM, support vector Machine; ANN, artificial neural network.

**Figure 6:**
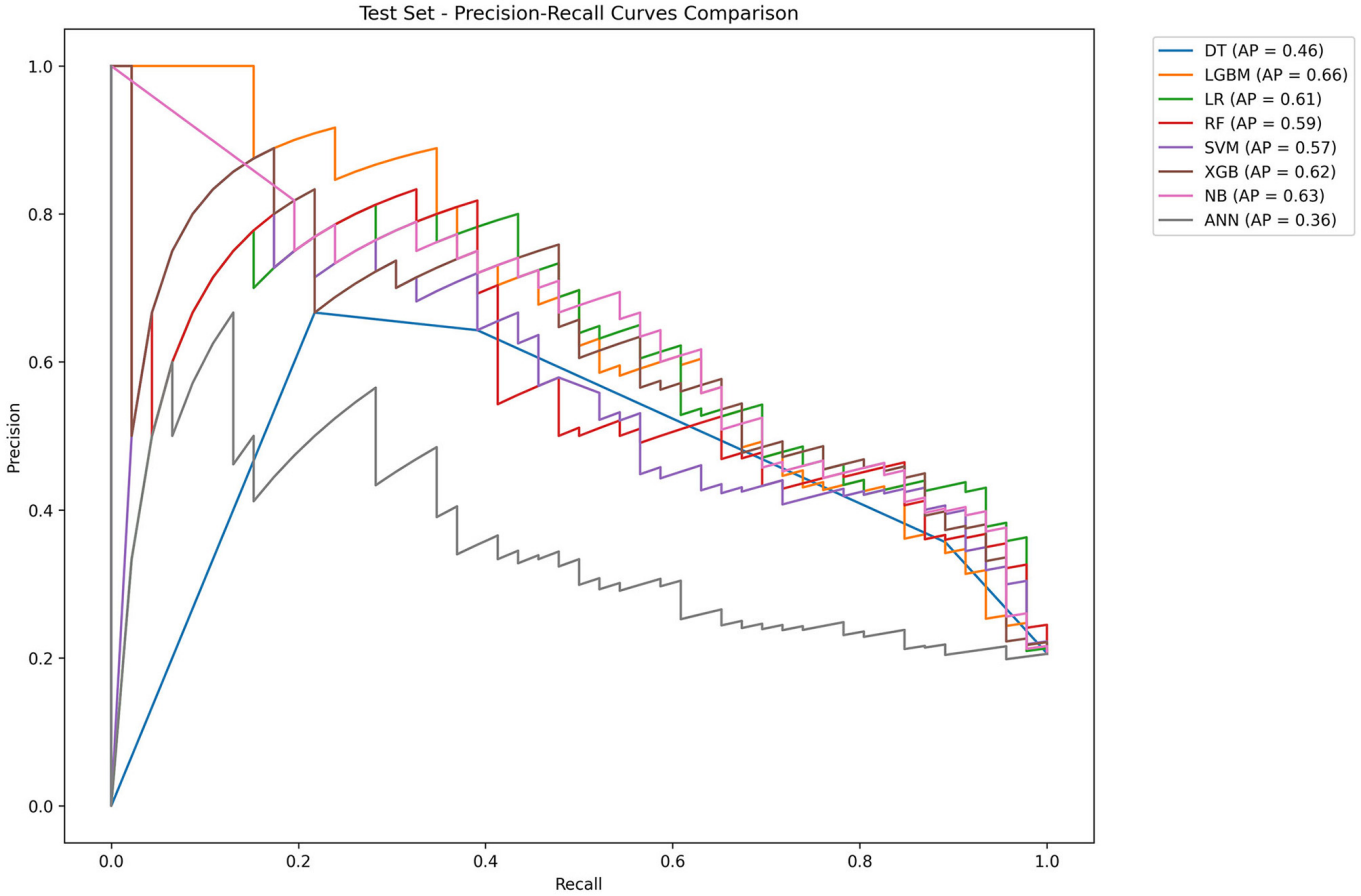
Precision–recall curves of eight machine learning models. LR, logistic regression; RF, random forest; LGBM, light gradient boosting machine; NB, naive bayes; DT, decision tree; XGBoost, extreme gradient boosting; SVM, support vector Machine; ANN, artificial neural network.

Table 2 and Figure 4 showed the metrics for each model in predicting mortality. The LR model outperformed others with the highest accuracy of 0.8545 and a higher recall of 0.7826. The Jordan index of 0.7291, calibration slope of 0.7623, and Brier score of 0.1650 all indicated better model performances. On the training set, both LGBM (AUC = 0.9907) and XGBoost (AUC = 0.9780) achieved near-perfect AUC scores approaching 1.0, whereas their performance declined markedly on the test set (AUC = 0.8396 and 0.8510), indicating potential overfitting. In contrast, the LR model demonstrated relatively excellent and stable performance across both the training and test sets, achieving the highest prediction performance on the test set (AUC = 0.8646), as is shown in Figure 5. The precision-recall curve is a visualization tool used to evaluate the trade-off relationship between the precision and recall of a classification model at different thresholds. Figure 6 showed that this LR model algorithm has high classification precision and recall rate. Therefore, the LR model was finally selected to predict the mortality rate of spontaneous SAH patients in the ICU.

**Table 2:** Evaluation of different machine learning models.

**Models**	**Sets**	**AUC**	**Accuracy**	**Precision**	**Recall**	**Brier score**	**Jordan index**	**Calibration** **slope**
ANN	Training set	0.6993	0.7904	0.5752	0.3171	0.1888	0.3985	0.4228
	Test set	0.6373	0.7812	0.4571	0.3478	0.1997	0.2745	0.3535
DT	Training set	0.8482	0.6934	0.4219	0.9220	0.1491	0.6964	0.8394
	Test set	0.7938	0.6473	0.3565	0.8913	0.171	0.5876	0.7129
LGBM	Training set	0.9907	0.9398	0.7938	0.9951	0.0574	0.9814	1.2931
	Test set	0.8396	0.7902	0.4921	0.6739	0.1372	0.6793	0.6881
LR	Training set	0.8451	0.8681	0.4949	0.7171	0.1613	0.6902	0.8512
	Test set	0.8646	0.8245	0.4444	0.7826	0.1650	0.7291	0.7623
NB	Training set	0.8658	0.8161	0.6075	0.5512	0.1642	0.7317	0.2404
	Test set	0.8577	0.8259	0.5660	0.6522	0.1579	0.7154	0.7689
RF	Training set	0.8969	0.7949	0.5344	0.7951	0.1415	0.7939	1.3670
	Test set	0.8448	0.7545	0.4483	0.8478	0.1590	0.6895	1.1009
SVM	Training set	0.8485	0.7670	0.4938	0.7756	0.1246	0.6969	1.0644
	Test set	0.8340	0.7366	0.4253	0.8043	0.1260	0.6681	0.5259
XGBoost	Training set	0.9780	0.9320	0.9337	0.7561	0.0549	0.9559	1.2734
	Test set	0.8510	0.8348	0.6154	0.5217	0.1176	0.7020	0.6313

**Figure 7:**
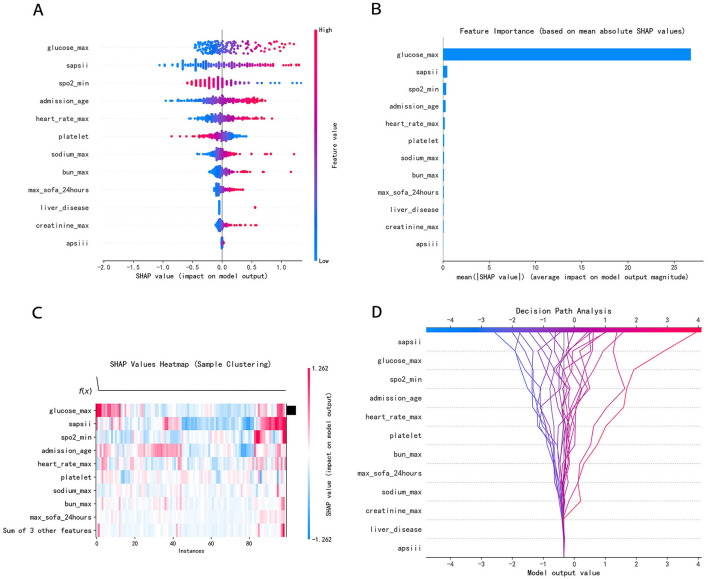
**(A)** SHAP summary dot plot; **(B)** SHAP summary bar plot; **(C)** SHAP heatmap plot; **(D)** SHAP decision plot. **(A)** Color denotes the feature value–red denotes a value that is greater, and blue denotes a value that is lower. The more dispersed the points of the graph represent, the greater the impact of the feature on the model. **(B)** The average SHAP values were calculated and ranked in descending order, with each row representing a distinct feature and the horizontal axis indicating the magnitude of its SHAP value. **(C)** Rows represent features, and columns represent samples. The color intensity reflects the magnitude of feature values on the model output (SHAP value). Red has a greater influence, while blue has a lesser influence. **(D)** The features were listed in order of decreasing importance, based on their cumulative SHAP values across the plotted observations. Every line converges to a single point at −0.323.

To identify the most influential features, we employed the SHAP value analysis, an interpretable method widely used in medical research. By visualizing the SHAP values of all features across the entire dataset, we were able to discern overarching patterns and relationships within the data. As illustrated in the SHAP summary plots (Figures 7A, B), we evaluated the contributions of 12 selected features to the model performance. We further transformed the SHAP value matrix into a heatmap (Figure 7C) to visualize the feature contributions at the individual sample level. This visualization provided a granular view of how each feature contributed to the prediction for every sample. In Figure 7D, the decision plot depicted the decision-making process for each participant. Every line converges to a single point at −0.323.

## 4 Discussion

Spontaneous SAH is an acute cerebrovascular disease with a life-threatening neurological condition, which imposes a heavy burden on individuals, families, and even society (19). Once the disease occurs, most patients will be sent to the ICU for rescue and treatment. However, many patients still have poor prognosis or even death (19). Currently, the majority of studies are limited to the impact of a single conventional indicator or factor on mortality, and they lack an analysis of multiple causes of death. There are some studies that have reported that gender, WFNS class, APACHE II score, IL-6, Hunt and Hess grade, troponin I, white blood cell count, and electrocardiographic abnormalities are associated with spontaneous SAH (20).

We extracted relevant indicators from the MIMIC-IV database as comprehensively as possible for machine learning. To refine the feature selection process, we applied collinearity analysis and LASSO regression, thereby effectively reducing the influence of multicollinearity on the selected features. Subsequently, multiple models were developed using various machine learning algorithms. These models were evaluated based on multiple parameters to ensure that the final model had better and more stable performance in predicting the outcome of death.

We found, quite interestingly, patients with hyperglycemia on admission had increased mortality most significantly. A retrospective analysis showed that admission hyperglycemia was associated with significantly increased mortality in critically ill patients with SAH (21). The causes of admission hyperglycemia, which could either be pre-existing diabetes mellitus or stress-induced hyperglycemia. Stress-induced hyperglycemia was an independent risk factor for pulmonary infection and death after intracranial hemorrhage (22). In clinical practice, blood glucose is an indicator that can be quickly obtained. In subsequent studies, we will focus on blood glucose to explore the mechanism of hyperglycemia on the death of spontaneous SAH patients. Rapidly detecting blood glucose and reducing hyperglycemia that may occur in the early stage of the disease are likely to become a potent measure to reduce the mortality rate of patients.

In our study, low SpO_2_ indicates increased mortality, which may be related to the lack of oxygen in patients. However, a study utilizing data from large ICU databases revealed a U-shaped relationship between SpO_2_ levels and mortality among patients (23). For patients with TBI and SAH, maintaining SpO_2_ at 94–96% will minimize the in-hospital mortality of patients. In the other study, the optimal range of SpO_2_ was 94% to 98% (24). The patients who were within the optimal range of SpO_2_ were associated with decreased hospital mortality (23). This finding is different from the results of our study. In the subsequent research, we can conduct a separate analysis of the relationship between SpO_2_ and mortality.

Regardless of whether baseline analysis or machine learning models were used, older age was associated with a higher risk of mortality. It is well-established that as patients grow older, their organ functions become increasingly susceptible to failure (25). Therefore, when elderly patients experience an acute disease, like spontaneous SAH, they will probably be at relatively high risk of death.

In terms of vital signs, patients with higher heart rates on ICU admission had a higher risk of death. Studies have shown that increased sympathetic nerve stimulation in patients with subarachnoid hemorrhage leads to increased heart rate and even arrhythmia, and severe sympathetic nerve stimulation can greatly increase the risk of cardiac arrest, thereby increasing mortality (26). Therefore, reducing the heart rate of patients within the normal range and correcting arrhythmia immediately on admission can reduce the mortality of patients.

The on-admission platelet count was found to be significant and predictive of patient outcome on discharge (27). In our study, spontaneous SAH patients with low platelet counts have an increased risk of death. According to the literature, thrombocytopenia has been identified as an independent risk factor for symptomatic vasospasm following aneurysmal subarachnoid hemorrhage (28). Additionally, thrombocytopenia is associated with an increased risk of bleeding, which in turn elevates the mortality risk among patients.

We analyzed serum sodium, potassium, and calcium levels and found that serum sodium may be associated with the risk of death, with higher sodium levels at ICU admission indicating a higher risk of death. A study found that high serum sodium levels are related to higher ICU and hospital mortality in patients with non-traumatic SAH (29). Increased intracranial pressure (ICP) impairs hypothalamic function, thereby resulting in electrolyte disturbances in the body (30). Therefore, timely identification and correction of electrolyte disturbances is critical to prevent permanent central nervous system damage. Elevated serum sodium levels are indicative of severe intracranial hemorrhage and significant neurological dysfunction, potentially guiding clinicians to promptly initiate pharmacological interventions or surgical procedures aimed at reducing ICP.

Impaired kidney or liver function was also associated with high mortality (31). Several studies have shown that the underlying mechanism may involve renal failure leading to severe electrolyte disturbances and acid-base imbalances (32), as well as liver failure resulting in coagulopathy, which increases the risk of bleeding (33). This suggests that for patients with spontaneous SAH, early initiation of liver and renal protection treatment may be conducive to reducing the mortality of patients.

Several grading systems have been used to predict the outcome of critically ill patients (34). SAPS II score, APS III score, and SOFA score are related to the health status and organ function of patients. The higher the score, the worse the health status of the patients and the higher the risk of death (35).

Furthermore, the best performance of a model under the current methodology reflects the effectiveness of the selection process rather than an inherent advantage of the model itself. While LR remains remarkably competitive in the wave of Artificial Intelligence (AI), no single model is universally superior—optimal performance is fundamentally contingent on careful matching of algorithmic strengths to the specific data characteristics and specific research constraints at hand.

The study possesses several notable strengths: (1) The data were sourced from a large, publicly accessible database on the Internet, ensuring reliability and representativeness. (2) Collinearity analysis was used for feature selection, enhancing the robustness of the model. (3) Multiple machine learning algorithms were used to construct models capable of ranking feature importance. (4) The final 12 selected features are readily available clinically. There is no analysis of the combined effects of these features on SAH.

Additionally, this study had several limitations: (1) The study lacks external validation. We have initiated the collection of relevant data from our hospital and plan to conduct further research upon reaching the target sample size. (2) This study captured laboratory indicators only on the first day of ICU admission, lacking dynamic monitoring of these indicators over time. (3) In future follow-up studies, targeting aneurysmal SAH exclusively could help exclude the influence of etiology on the results, thereby enhancing the specificity of the findings.

## 5 Conclusion

Our study develops an interpretable machine learning model to predict the risk factors and mortality in patients with spontaneous SAH. We selected the best-performing model among the eight models, namely the LR model. This model incorporates 12 features. Finally, SHAP was used to interpret the model to improve the interpretability of the model. This study may facilitate the early identification of mortality risk in patients with spontaneous SAH, thereby enabling timely intervention. Moreover, it can assist clinicians in optimizing patient management under resource constraints, thus reducing mortality risk and improving clinical outcomes.

## Data Availability

The datasets presented in this study can be found in online repositories. The names of the repository/repositories and accession number(s) can be found in the article.

## Supplementary material

Supplementary Figure S1 Collinearity analysis. VIF, variance inflation factor. VIF = 1: no multicollinearity, VIF between 1 and 10: moderate multicollinearity, VIF > 10: serious multicollinearity. Blue bars: the retained features. Green bars: the removed features.

Supplementary Table S1 The missing data of this study.
