# Supplementary figures and images for: Machine learning models for mortality prediction in patients with spontaneous subarachnoid hemorrhage following ICU treatment
Source: Front Neurol. 2025 Sep 17;16:1648353. doi: 10.3389/fneur.2025.1648353 (PMC12485233; doi:10.3389/fneur.2025.1648353)

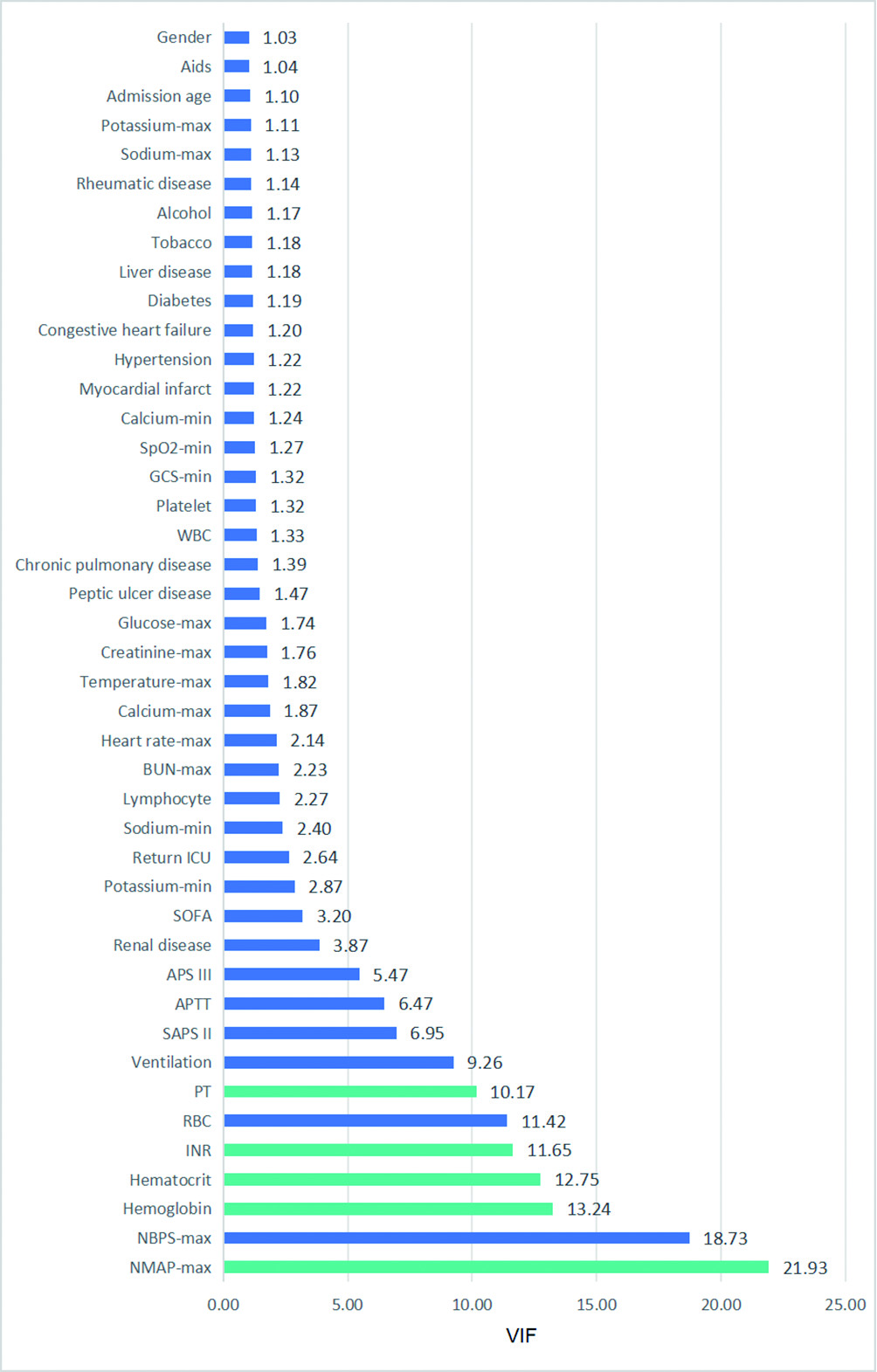

Supplement: Supplementary Figure S1 — Collinearity analysis. VIF, variance inflation factor. VIF = 1: no multicollinearity, VIF between 1 and 10: moderate multicollinearity, VIF > 10: serious multicollinearity. Blue bars: the retained features. Green bars: the removed features. [file Image_1.jpeg]
