# Supplementary material for: Machine learning models for mortality prediction in patients with spontaneous subarachnoid hemorrhage following ICU treatment
Source: Front Neurol. 2025 Sep 17;16:1648353. doi: 10.3389/fneur.2025.1648353 (PMC12485233; doi:10.3389/fneur.2025.1648353)
Supplement: Supplementary Table S1 — The missing data of this study. [file Table_1.docx]

| Supplementary Table S1. The missing data of this study. | | |
| --- | --- | --- |
| Variable | Missing Count | Missing Percentage |
| APTT | 154 | 0.1374 |
| PT | 137 | 0.1222 |
| INR | 109 | 0.0972 |
| RBC | 70 | 0.0624 |
| WBC | 70 | 0.0624 |
| Hemoglobin | 67 | 0.0598 |
| Platelet | 66 | 0.0589 |
| Hematocrit | 65 | 0.0580 |
| Calcium_min | 64 | 0.0571 |
| Calcium_max | 64 | 0.0571 |
| Glucose_max | 41 | 0.0366 |
| Sodium_max | 33 | 0.0294 |
| Sodium_min | 33 | 0.0294 |
| Potassium_max | 33 | 0.0294 |
| Potassium_min | 33 | 0.0294 |
| Urineoutput | 28 | 0.0250 |
| Creatinine_max | 16 | 0.0143 |
| Bun_max | 16 | 0.0143 |
| Temperature_max | 14 | 0.0125 |
| NMAP _max | 4 | 0.0036 |
| GCS_min | 3 | 0.0027 |
| NBPS _max | 3 | 0.0027 |
| Max_SOFA_24hours | 2 | 0.0018 |
| Heart_rate_max | 2 | 0.0018 |
| SpO_2__min | 2 | 0.0018 |

Abbreviations: PT: prothrombin time; APTT: activated partial thromboplastin time; INR: international normalized ratio; RBC: red blood cell count; WBC: white blood cell count; BUN: blood urea nitrogen; NBPS: non-invasive blood pressure systolic; NMAP: non-invasive mean arterial pressure; GCS: Glasgow coma scale; SOFA: sequential organ failure assessment; SpO_2_: saturation of pulse oxygen.
